# The Relationships Between MASLD, Extrahepatic Multimorbidity, and All-Cause Mortality in the UK Biobank Cohort

**DOI:** 10.1210/clinem/dgaf342

**Published:** 2025-06-10

**Authors:** Qi Feng, Chioma N Izzi-Engbeaya, Andrea D Branch, Benjamin H Mullish, Pinelopi Manousou, Mark Woodward

**Affiliations:** The George Institute for Global Health (UK), School of Public Health, Faculty of Medicine, Imperial College London, London W12 7RZ, UK; Section of Investigative Medicine and Endocrinology, Department of Metabolism, Digestion and Reproduction, Faculty of Medicine, Imperial College London, London W12 0NN, UK; Department of Medicine, Division of Liver Diseases, The Icahn School of Medicine at Mount Sinai, New York, NY 10029, USA; Division of Digestive Diseases, Department of Metabolism, Digestion and Reproduction, Faculty of Medicine, Imperial College London, London W12 0NN, UK; Department of Hepatology, St Mary's Hospital, Imperial College Healthcare NHS Trust, London W2 1NY, UK; Division of Digestive Diseases, Department of Metabolism, Digestion and Reproduction, Faculty of Medicine, Imperial College London, London W12 0NN, UK; Department of Hepatology, St Mary's Hospital, Imperial College Healthcare NHS Trust, London W2 1NY, UK; The George Institute for Global Health (UK), School of Public Health, Faculty of Medicine, Imperial College London, London W12 7RZ, UK; The George Institute for Global Health (Australia), University of South New Wales, Sydney, NSW 2000, Australia

**Keywords:** MASLD, NAFLD, multimorbidity, all-cause mortality, UK Biobank, long-term conditions, sexual dimorphism

## Abstract

**Context:**

Metabolic dysfunction–associated steatotic liver disease (MASLD) affects one third of the world's population, but its associations with extrahepatic multimorbidity and mortality remain unclear.

**Objective:**

This study aimed to estimate the impact of MASLD, with and without multimorbidity, on all-cause mortality.

**Methods:**

We analyzed data from the UK Biobank. MASLD was identified as a fatty liver index ≥60 and presence of cardiometabolic risk factors. Multimorbidity was defined as ≥2 of the long-term conditions (LTCs) in a prespecified list of 47 extrahepatic conditions. Hazard ratios (HRs) from adjusted Cox models quantified the association between MASLD, multimorbidity and all-cause mortality.

**Results:**

Of the 438 840 participants, 131 020 (29.9%) had MASLD at baseline. The participants with MASLD at baseline had a higher prevalence of multimorbidity than those without (21.3% vs 14.4%). In addition to cardiometabolic risk factors, MASLD was strongly associated with several LTCs, particularly metabolic, cardiovascular, cancers, kidney, mental/behavioral, and respiratory diseases. During a median follow-up of 13 years, MASLD was associated with higher mortality (HR 1.16; 95% CI 1.13, 1.19), with stronger associations in females and in those with low LTC counts (≤3 LTCs). Each additional LTC at baseline was associated with 30% and 38% higher mortality in MASLD (HR 1.30; 1.29, 1.32) and non-MASLD (HR 1.38; 1.37, 1.40) populations, respectively. Among the 47 LTCs, 16 were associated with increased mortality in people with MASLD.

**Conclusion:**

Those with MASLD exhibited a higher prevalence of extrahepatic multimorbidity and a 16% higher rate of mortality than those without, underscoring the impact of liver steatosis on mortality and highlighting the need to target LTCs to improve outcomes and reduce health care burdens.

Metabolic dysfunction–associated steatotic liver disease (MASLD), the replacement term for nonalcoholic fatty liver disease (NAFLD), is defined as the presence of liver steatosis along with 1 or more case-defining cardiometabolic risk factors (obesity, diabetes, hypertension, and dyslipidemia) in the absence of excessive alcohol consumption or other established chronic liver diseases (1). MASLD consists of a spectrum of hepatic conditions, ranging from simple steatosis to steatohepatitis, and can progress to fibrosis, cirrhosis, and hepatocellular carcinoma (1, 2). MASLD, the most common liver condition, has emerged as a significant public health concern globally, with a prevalence of 32.4% (2-4). The high prevalence of MASLD is associated with an increased risk of hepatic morbidity and mortality, as well as a substantial economic burden (5, 6).

Multimorbidity is defined here as the coexistence of 2 or more long-term conditions (LTCs) that are on a specific predefined list (7). Multimorbidity increases the severity and progression rate of many adverse health outcomes, such as cognitive decline (8), cancers (9), cardiovascular disease (10), reduced health-related quality of life (11), and mortality (12). While the burden of multimorbidity has been studied in the general population (9, 13) and in specific groups, such as people with diabetes (14) and with stroke (15), its implications in individuals with MASLD have not been comprehensively studied.

MASLD is considered to be the hepatic manifestation of metabolic syndrome (16) and is associated with diabetes (17), cardiovascular disease (18), extrahepatic cancers (19), and chronic kidney disease (CKD) (20). Consequently, individuals with MASLD are likely to be at increased risk for extrahepatic multimorbidity. However, previous research investigating multimorbidity in patients with MASLD often focused on a narrow range of conditions (21, 22). For instance, an Italian study described multimorbidity in 95 patients with diabetes and NAFLD but only considered 16 LTCs (23). A study in Israel examined 200 000 patients with NAFLD but included only 14 LTCs, half of which were metabolic disorders (24). Therefore, a formal investigation of the relationship between MASLD and multimorbidity defined using a more extensive list of LTCs is warranted.

In the UK, liver-related mortality has increased 4-fold since the 1970s, positioning liver disease as the third most common cause of premature death (25). MASLD has been an increasingly important contributor to liver-related mortality. Although it is a distant second to alcohol-related liver disease, MASLD surpasses viral hepatitis, autoimmune or metabolic liver diseases, and other causes in the UK (26). MASLD has become one of the most common indications for liver transplantation (27). To understand the clinical consequences of metabolic dysfunction, it is crucial to understand MASLD, extrahepatic multimorbidity, and their interactions with health outcomes.

Accordingly, we aimed to describe the prevalence of multimorbidity, in individuals with MASLD, and to quantify the impact of MASLD, in conjunction with the degree of multimorbidity, on all-cause mortality in a large UK-based study.

## Materials and Methods

We used data from the UK Biobank, a population-based cohort of half a million participants aged between 40 and 70 years old recruited between 2006 and 2010 (28). At baseline assessment, data were collected on sociodemographic characteristics, lifestyle, health status, medication use, environmental factors, anthropometry, and physical measures. Biological samples including blood, saliva, and urine were collected. Participants were prospectively followed up via linkage to national death registries, cancer registries, and hospitalization data (28). UK Biobank was approved by the North-West Multi-centre Research Ethics Committee, the National Information Governance Board for Health and Social Care in England and Wales, and the Community Health Index Advisory Group in Scotland. All participants provided informed consent.

We used the definition proposed by Rinella et al to identify individuals with MASLD, which consists of liver steatosis, at least 1 cardiometabolic risk factor, and low alcohol consumption (<20 [female]/30 [male] g/day) (29). We used the fatty liver index (FLI) ≥60% as an indicator of liver steatosis. FLI is a score representing hepatic steatosis levels, calculated from the body mass index (BMI), waist circumference, triglycerides, and g-glutamyl transferase (30). The cutoff value of 60% has been used previously to define steatosis (5, 6). The accuracy of FLI in identifying individuals with steatosis has been validated (6). We further identified individuals with any of the following cardiometabolic risk factors: obesity (BMI ≥ 25 kg/m^2^ AND/OR waist circumference >94 cm [male], >90 cm female]), diabetes (glycated hemoglobin [HbA1c] ≥48 mmol/mol AND/OR diagnosis of type 2 diabetes AND/OR on treatment for type 2 diabetes), hypertension (systolic blood pressure [BP] ≥130 AND/OR diastolic BP ≥85 mmHg AND/OR on antihypertensive drug treatment or diagnosis of hypertension), high triglycerides (TGs) (plasma TG ≥1.70 mmol/L AND/OR on lipid-lowering treatment), and low high-density lipoprotein (HDL) cholesterol (HDL cholesterol ≤1.0 mmol/L [male], ≤1.3 mmol/L [female] AND/OR on lipid-lowering treatment) (29).

We excluded women who were pregnant at baseline, and people who had missing data for calculating FLI or defining MASLD. We also excluded people with alcohol-related liver disease, metabolic dysfunction– and alcohol-related liver disease, or other chronic liver conditions (including viral hepatitis, liver fibrosis, liver cirrhosis, hepatocellular carcinoma, hemochromatosis, Wilson disease, biliary cirrhosis, autoimmune hepatitis, primary sclerosing cholangitis, toxic liver disease, and Budd–Chiari syndrome). For alcohol-related liver disease and metabolic dysfunction– and alcohol-related liver disease, we used the definition proposed by Rinella et al (29), whereas other chronic liver disease was ascertained via self-report and hospitalization data; the code lists for these conditions are presented in the supplemental material (31).

### Exposure

We used the self-report data and hospitalization records for phenotyping multimorbidity. We defined multimorbidity as having 2 or more LTCs from a prespecified list. We based our list of LTCs from those identified in a 3-round Delphi study of 25 public participants and 150 health care professionals (including clinicians, researchers, and policy makers) in 2021 (32). The criteria for including these LTCs included their impacts on risk of death, quality of life, frailty, physical disability, mental health, and treatment burden. Among the conditions identified in this Delphi study, we combined solid organ cancers, metastatic cancers, melanoma, and treated cancer requiring surveillance into 1 broad solid organ cancer category, and removed post-acute Covid-19, chronic Lyme disease, and 2 liver conditions (hepatocellular carcinoma and chronic liver disease). Since MASLD is our index disease, we excluded MASLD and its 5 associated cardiometabolic risk factors (obesity, hypertension, diabetes, high TG, and low HDL cholesterol) from the list. After these modifications, this study considered 47 LTCs for multimorbidity (supplemental material (31)). These LTCs covered extrahepatic cancers, cardiovascular, metabolic, endocrinological, respiratory, digestive, renal, mental/behavioral, and congenital conditions. We measured the number of LTCs for each individual at baseline visit and annually for up to 10 years of follow-up, enabling us to examine the trajectory of LTC accumulation over time, and compared patterns between people with and without MASLD.

### Outcome

Our outcome was all-cause mortality within the follow-up period of the study, confirmed via death registry records. Participants were censored at the date of death or the last date of follow-up (November 30, 2022), whichever occurred first.

#### Covariates

A participant's region was determined by the location of the assessment center they attended at baseline. Ethnicity was classified into Asian, Black, White, and mixed/others. The Townsend Deprivation Index is a postcode-derived measure used to designate socioeconomic status. Educational attainment was categorized as below secondary, lower secondary, upper secondary, vocational training, and higher education. Lifestyle factors considered were self-reported current smoking (current, previous, and never smoker), alcohol consumption, and physical activity level. Alcohol consumption was assessed via self-reported weekly or monthly intake of red wine, white wine, champagne, beer, spirits, fortified wine, and other alcoholic drinks; the consumption was converted to standard UK alcohol units and further to alcohol grams and summed up to derive the average daily alcohol consumption (g/d) (33). Physical activity level was measured with the International Physical Activity Questionnaire and centrally processed by the UK Biobank; individuals were categorized into low, moderate, and high levels, based on the frequency, duration, and intensity of their physical activities. Systolic and diastolic BP were measured by trained staff twice within a few minutes apart and the averages of the 2 readings were used in analyses. Blood biochemistry markers were measured at a central laboratory by the UK Biobank using the blood sample collected at baseline, including TG, HDL cholesterol, low-density lipoprotein cholesterol, glucose, HbA1c, and hemoglobin levels. Fibrosis severity was measured with FIB4 score, using the cutoff values of 1.3 and 2.67 (for people aged <65 years) and 2.0, 2.67 (for people aged ≥65 years) for low, intermediate, and high risk, respectively (34). For all the categorical covariates, answers of “unknown,” “do not know,” “prefer not to say,” were combined into 1 “unknown” category.

### Statistical Analysis

The baseline characteristics were summarized using mean with SD or median with interquartile interval, as appropriate, and frequency with percentage, stratified by MASLD status and the number of LTCs (0, 1, and ≥2 LTCs).

We used bar charts to demonstrate the distribution of the baseline number of LTCs stratified by MASLD status, sex, and age groups (<50, 50-59, ≥60 years). We calculated the prevalence (per 1000) for each LTC and ranked these factors in people with and without MASLD separately. We fitted logistic regression models, adjusted for sex, age, education, and Townsend Deprivation Index (in fifths), to estimate prevalence odds ratios (ORs) and their 95% CIs relating MASLD status to each LTC cross-sectionally; *P* values were adjusted for multiple testing using the false discovery rate method (35). To illustrate the trajectory of annual prevalence of multimorbidity (≥2 LTCs) during follow-up, we made line plots stratified by MASLD status and highlighted the difference between the 2 groups at each time point. The prevalence of multimorbidity at each follow-up time point was calculated after excluding individuals who had died by that time point.

Cox proportional hazard regression models were used to assess the association between having MASLD and all-cause mortality, expressed as hazard ratio (HR) with 95% CI. Models were stratified by region and age group, and adjusted for sex, ethnicity, education, Townsend Deprivation Index (in fifths), physical activity level, smoking status, alcohol consumption, and LTC counts. The proportional hazard assumption was examined by scaled Schoenfeld residuals, and no evidence was observed for its violation. This analysis was repeated in people with different baseline LTC counts (0, 1, 2, 3, 4, ≥5), thus enabling us to assess the effect of additionally having MASLD on all-cause mortality, across a range of baseline LTC counts. We also evaluated the association between the baseline number of LTCs and all-cause mortality with similarly adjusted Cox models. The number of LTCs was fitted as a categorical (0, 1, 2, 3, 4, and ≥5 LTCs) and as a continuous (per each additional LTC) variable in separate models. We assessed the association of multimorbidity separately in MASLD and non-MASLD cohorts, and compared the 2 HRs using the ratio of hazard ratios (RHR) with its 95% CI (36). In sensitivity analysis, we (1) adjusted the Cox models additionally for the 5 cardiometabolic risk factors; (2) excluded the first 2 years of follow-up to correct for reverse causation; (3) modeled LTC number as a time-varying variable in the Cox model to reflect its trajectory during follow-up; and (4) applied an alternative definition of liver steatosis based on a combination of high hepatic steatotic index (HSI ≥36) (37). The association between LTC numbers and all-cause mortality was also assessed in subgroups stratified by age group, Townsend Deprivation Index (in fifths), education, smoking status, physical activity, BMI levels, diabetes status, and fibrosis severity.

We assessed the association between cardiometabolic risk factors and mortality, using people without the risk factor under examination as the reference group, in people with and with MASLD separately. We assessed the associations between specifically having any of the 47 LTCs and all-cause mortality, using people without any LTC as the reference group, in people with MASLD.

All analyses were conducted in R software and we applied a correction for multiple comparisons (35). This study is compliant with the Strobe checklist for cohort study.

## Results

### Participants and Baseline Characteristics

**Figure 1.**
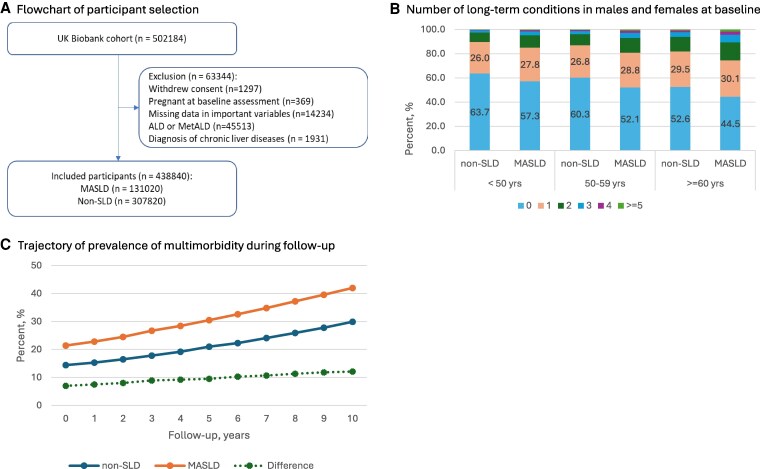
The flowchart of participants selection and the number of long-term conditions in people with and without MASLD, stratified by sex and age group. (A) Flowchart of participant selection. (B) Number of long-term conditions in males and females at baseline. (C) Trajectory of prevalence of multimorbidity during follow-up. Abbreviations: ALD, alcohol-related liver disease; MASLD, metabolic dysfunction-associated steatotic liver disease; MetALD, metabolic dysfunction– and alcohol-related liver disease; SLD, steatotic liver disease.

Among 438 840 eligible individuals (mean age 56.5 [SD 8.1] years, 42.2% males), we identified 131 020 (29.9%) individuals with MASLD (mean age 57.4 [SD 7.9], 58.2% males) (Fig. 1). MASLD prevalence was higher in males (41.2%) than females (21.6%) and in older people (24.5%, 29.7%, and 32.0% for people <50, 50-59, ≥60 years, respectively).

Overall, at baseline, 28.2% of participants had 1 of the designated LTCs, and 16.4% had ≥2 LTCs. The prevalence of having ≥2 LTCs was slightly higher in females (17.2%) than males (15.4%), in those with MASLD (21.3%) than in people without (14.4%), and increased with age (11.5%, 14.8%, and 20.4% for those <50, 50-59, and ≥60 years, respectively). Among people with MASLD, the prevalence of having ≥2 LTCs was higher in those with diabetes than in those without (27.8% vs 18.3%), and higher in those with high FIB4 scores than in those with low or intermediate levels (20.8%, 21.8% vs 29.9%).

During follow-up, the prevalence of multimorbidity among people without MASLD increased from 14.4% at baseline to 21.0% at year 5 and 29.9% at year 10. Among those with MASLD, the corresponding increase was from 21.4% at baseline to 30.5% and 42.0%, respectively. The difference in prevalence between the 2 groups widened slightly from 7.0% at baseline to 9.5% at year 5 and 12.1% at year 10 (Fig. 1).

Individuals with MASLD were more likely than those without to be of older age, male, more deprived, less educated, less physically active, and to have higher BMI, waist circumference, BPs, TGs, glucose, and HbA1c, and to have lower HDL (Table 1). The 3 most prevalent cardiometabolic risk factors were obesity (98.9%), hypertension (80.3%), and high TGs (72.6%) for people with MASLD, and hypertension (61.2%), obesity (55.9%), and low HDL cholesterol (30.1%) for people without MASLD. Among people with MASLD, 3.2%, 19.2%, 35.2%, 29.5%, and 12.9% had 1, 2, 3, 4, and 5 cardiometabolic risk factors, respectively. Among people without MASLD, 12.7%, 28.2%, 31.6%, 18.2%, 7.5%, and 1.8% had 0, 1, 2, 3, 4, and 5 cardiometabolic risk factors, respectively. People with more LTCs were more likely to be older, more socioeconomically deprived, less educated, less physically active, and exhibited higher BMI, waist circumference, and HbA1c. As expected, each of the 5 MASLD-defining cardiometabolic risk factors was more prevalent in the MASLD cohort than the non-MASLD cohort. Table S1 provides more details about baseline characteristics (31).

**Table 1.** Baseline characteristics of participants with and without MASLD, stratified by number of long-term conditions at baseline

	No long-term condition		1 long-term condition		2 or more long-term conditions		Total
	Non-SLD	MASLD	Non-SLD	MASLD	Non-SLD	MASLD	
	n = 178 357	n = 64 823	n = 85 262	n = 38 312	n = 44 201	n = 27 885	n = 438 840
Sex, male	66 167 (37.1%)	40 767 (62.9%)*^a^*	28 414 (33.3%)	21 333 (55.7%)*^a^*	14 374 (32.5%)	14 175 (50.8%)*^a^*	185 230 (42.2%)
Age, years	55.4 (8.2)	56.6 (7.9)*^a^*	56.6 (8.2)	57.7 (7.8)*^a^*	58.1 (8.0)	59.1 (7.5)*^a^*	56.5 (8.1)
Townsend Deprivation Index							
1st fifth (least deprived)	38 336 (21.5%)	12 558 (19.4%)*^a^*	18 009 (21.1%)	6982 (18.2%)*^a^*	8355 (18.9%)	4086 (14.7%)*^a^*	88 326 (20.1%)
5th fifth (most deprived)	31 181 (17.5%)	13 685 (21.1%)	15 488 (18.2%)	8918 (23.3%)	9996 (22.6%)	8320 (29.8%)	87 588 (20.0%)
Education, higher education	66 587 (37.3%)	18 240 (28.1%)*^a^*	29 978 (35.2%)	10 026 (26.2%)*^a^*	13 307 (30.1%)	5942 (21.3%)*^a^*	144 080 (32.8%)
Ethnicity, White	167 842 (94.1%)	59 845 (92.3%)*^a^*	80 866 (94.8%)	35 899 (93.7%)*^a^*	41 951 (94.9%)	26 175 (93.9%)*^a^*	412 578 (94.0%)
Smoking, never	107 463 (60.3%)	36 154 (55.8%)*^a^*	49 133 (57.6%)	20 163 (52.6%)*^a^*	22 892 (51.8%)	12 829 (46.0%)*^a^*	248 634 (56.7%)
Alcohol drinking, g/d	10.3 (19.0)	6.9 (16.0)*^a^*	9.1 (18.8)	5.1 (14.2)*^a^*	6.9 (17.3)	2.0 (11.9)*^a^*	12.2 (15.2)
Physical activity, high	63 798 (35.8%)	17 614 (27.2%)*^a^*	28 765 (33.7%)	9690 (25.3%)*^a^*	13 640 (30.9%)	6102 (21.9%)*^a^*	139 609 (31.8%)
ALT, log_10_ U/L	1.2 (0.3)	1.4 (0.2)*^a^*	1.2 (0.3)	1.4 (0.2)*^a^*	1.2 (0.3)	1.4 (0.2)*^a^*	1.3 (0.3)
AST, log_10_ U/L	1.3 (0.2)	1.4 (0.1)*^a^*	1.3 (0.2)	1.4 (0.1)*^a^*	1.3 (0.3)	1.4 (0.2)*^a^*	1.3 (0.2)
FIB4 score	—	1.3 (1.3)	—	1.3 (0.7)	—	1.4 (6.3)	1.3 (2.0)
BMI, kg/m^2^	25.0 (3.0)	31.4 (4.4)*^a^*	25.1 (3.1)	31.9 (4.6)*^a^*	25.3 (3.3)	32.7 (5.1)*^a^*	27.1 (4.7)
Waist circumference, cm	82.9 (9.4)	101.9 (9.7)*^a^*	83.3 (9.4)	102.6 (10.2)*^a^*	84.0 (9.7)	104.4 (11.1)*^a^*	89.0 (13.1)
Systolic blood pressure, mmHg	135.2 (18.6)	142.5 (17.4)*^a^*	135.1 (18.6)	141.5 (17.4)*^a^*	135.3 (18.9)	139.8 (17.7)*^a^*	137.1 (18.6)
Diastolic blood pressure, mmHg	80.5 (9.8)	86.0 (9.5)*^a^*	80.2 (9.8)	84.9 (9.6)*^a^*	79.7 (10.0)	83.3 (10.1)*^a^*	81.7 (10.0)
Triglyceride, mmol/L	1.1 (0.7)	2.2 (1.3)*^a^*	1.2 (0.7)	2.1 (1.3)*^a^*	1.2 (0.8)	2.1 (1.3)*^a^*	1.4 (1.0)
HDL cholesterol, mmol/L	1.4 (0.6)	1.1 (0.4)*^a^*	1.4 (0.6)	1.1 (0.4)*^a^*	1.3 (0.6)	1.1 (0.4)*^a^*	1.3 (0.6)
HbA1c, mmol/mol	33.8 (6.5)	36.5 (9.5)*^a^*	34.1 (6.7)	37.2 (10.0)*^a^*	34.8 (7.8)	38.7 (11.5)*^a^*	35.0 (8.1)
Hypertension	108 041 (60.6%)	52 652 (81.2%)*^a^*	52 413 (61.5%)	30 724 (80.2%)*^a^*	27 943 (63.2%)	21 801 (78.2%)*^a^*	293 574 (66.9%)
Obesity	96 588 (54.2%)	63 985 (98.7%)*^a^*	48 852 (57.3%)	37 914 (99.0%)*^a^*	26 674 (60.3%)	27 654 (99.2%)*^a^*	301 667 (68.7%)
Diabetes	19 668 (11.0%)	17 394 (26.8%)*^a^*	10 935 (12.8%)	12 112 (31.6%)*^a^*	7830 (17.7%)	11 403 (40.9%)*^a^*	79 342 (18.1%)
High triglyceride	41 605 (23.3%)	46 871 (72.3%)*^a^*	22 447 (26.3%)	27 820 (72.6%)*^a^*	13 868 (31.4%)	20 425 (73.2%)*^a^*	173 036 (39.4%)
Low HDL cholesterol	49 354 (27.7%)	27 731 (42.8%)*^a^*	26 658 (31.3%)	18 137 (47.3%)*^a^*	16 544 (37.4%)	15 289 (54.8%)*^a^*	153 713 (35.0%)

Number (number): mean (SD). Number (%): frequency (%).

Abbreviations: ALT, alanine aminotrasnferase; AST, aspartate aminotransferase; BMI, body mass index; HbA1c, glycated hemoglobin; HDL, high-density lipoprotein; LDL, low-density lipoprotein; MASLD, metabolic dysfunction–associated steatotic liver disease.

^*a*^Showing statistical difference between MASLD and non-SLD groups, using t test for normal continuous variables, Mann–Whitney U test for non-normal continuous variables, and chi-square test for categorical variables.

### Prevalence of Cardiometabolic Risk Factors and LTCs

The crude prevalence of each condition, stratified by MASLD and sex, is shown in Table S2 (31).

Overall, the top 6 most prevalent LTCs were similar in people with and without MASLD (Fig. S1 (31)), although rankings varied. The top 6 conditions were asthma, chronic respiratory disease (excluding asthma and chronic obstructive pulmonary disease [COPD]), thyroid disorder, depression, extrahepatic solid organ cancers, and osteoporosis in females without MASLD; asthma, thyroid disorder, chronic respiratory disease, depression, extrahepatic solid organ cancer, and ischemic heart disease (IHD) in females with MASLD; chronic respiratory disease, asthma, IHD, extrahepatic solid organ cancer, depression, and arrythmia in males without MASLD; and chronic respiratory disease, asthma, IHD, depression, extrahepatic solid organ cancer, and arrythmia in males with MASLD.

After adjusting for sex, age, education and Townsend Deprivation Index, individuals with MASLD were more likely to have 32 of the 47 LTCs, with the highest ORs for gout (2.83 [2.42, 3.32]), heart failure (2.24 [2.03, 2.46]), post-traumatic stress disorder (2.13 [1.68, 2.68]), osteoarthritis (1.98 [1.91, 2.06]), and bipolar disorder (1.90 [1.71, 2.12]). Overall, metabolic, mental/behavioral, cardiovascular, and renal conditions were more prevalent in patients with MASLD, while substance use disorder (0.83 [0.77, 0.89]), osteoporosis (0.63 [0.60, 0.67]), and eating disorder (0.42 [0.30, 0.59]) were less common in people with MASLD than in people without. Most ORs were higher for females than for males; for instance, the ORs for gout were 4.76 (3.12, 7.25) and 2.57 (2.16, 3.04), for stroke 1.66 (1.54, 1.80) and 1.41 (1.32, 1.51), for IHD 2.13 (2.03, 2.24) and 1.71 (1.65, 1.77), for COPD 1.34 (1.25, 1.43) and 1.16 (1.08, 1.24), for transient ischemic attack 1.52 (1.24, 1.85) and 1.26 (1.06, 1.49), for females and males, respectively. Some LTCs were significantly associated with MASLD in females but not in males, such as multiple sclerosis (1.18 [1.03, 1.36] vs 1.01 [0.82, 1.23]) and extrahepatic solid organ cancers (1.09 [1.05, 1.14] vs 1.05 [0.99, 1.10]) (Table 2).

**Table 2.** Adjusted prevalence odds ratios (95% CI) comparing MASLD with non-SLD on long-term conditions at baseline, by sex

	All			Females			Males		
	Condition	OR (95% CI)	*P* value*^a^*	Condition	OR (95% CI)	*P* value*^a^*	Condition	OR (95% CI)	*P* value*^a^*
1	Gout	2.83 (2.42, 3.32)	<.01	Autism	17.23 (1.75, 169.7)	.02	Gout	2.57 (2.16, 3.04)	<.01
2	Heart failure	2.24 (2.03, 2.46)	<.01	Gout	4.76 (3.12, 7.25)	<.01	Heart failure	2.13 (1.91, 2.39)	<.01
3	PTSD	2.13 (1.68, 2.68)	<.01	HIV/AIDS	2.78 (1.65, 4.66)	<.01	PTSD	1.92 (1.39, 2.65)	<.01
4	Osteoarthritis	1.98 (1.91, 2.06)	<.01	Heart failure	2.49 (2.09, 2.98)	<.01	Venous thromboembolism	1.72 (1.56, 1.90)	<.01
5	Bipolar disorder	1.90 (1.71, 2.12)	<.01	PTSD	2.46 (1.77, 3.43)	<.01	Ischemic heart disease	1.71 (1.65, 1.77)	<.01
6	Ischemic heart disease	1.86 (1.81, 1.91)	<.01	Chromosomal abnormalities	2.45 (1.23, 4.87)	.02	Osteoarthritis	1.67 (1.58, 1.77)	<.01
7	Venous thromboembolism	1.85 (1.73, 1.98)	<.01	Osteoarthritis	2.26 (2.15, 2.37)	<.01	Bipolar disorder	1.61 (1.37, 1.90)	<.01
8	Chromosomal abnormalities	1.81 (1.05, 3.11)	.05	Bipolar disorder	2.19 (1.90, 2.52)	<.01	Chronic kidney disease	1.59 (1.42, 1.79)	<.01
9	Schizophrenia	1.65 (1.45, 1.88)	<.01	Schizophrenia	2.18 (1.79, 2.66)	<.01	Epilepsy	1.45 (1.32, 1.58)	<.01
10	Vision impairment	1.64 (1.52, 1.78)	<.01	Ischemic heart disease	2.13 (2.03, 2.24)	<.01	Depression	1.44 (1.37, 1.50)	<.01
11	Chronic kidney disease	1.58 (1.45, 1.73)	<.01	Vision impairment	2.10 (1.87, 2.37)	<.01	Hearing impairment	1.44 (1.27, 1.63)	<.01
12	Anemia	1.58 (1.32, 1.90)	<.01	Venous thromboembolism	1.97 (1.81, 2.16)	<.01	Stroke	1.41 (1.32, 1.51)	<.01
13	Depression	1.57 (1.52, 1.61)	<.01	Paralysis	1.96 (1.63, 2.36)	<.01	Vision impairment	1.38 (1.25, 1.53)	<.01
14	Thyroid disorder	1.53 (1.49, 1.58)	<.01	Anemia	1.90 (1.53, 2.38)	<.01	Schizophrenia	1.37 (1.16, 1.62)	<.01
15	Epilepsy	1.52 (1.43, 1.62)	<.01	Depression	1.67 (1.61, 1.73)	<.01	Thyroid disorder	1.37 (1.28, 1.46)	<.01
16	Paralysis	1.52 (1.34, 1.73)	<.01	Stroke	1.66 (1.54, 1.80)	<.01	Arrythmia	1.36 (1.29, 1.43)	<.01
17	Stroke	1.51 (1.43, 1.59)	<.01	Peptic ulcer disease	1.63 (1.29, 2.06)	<.01	Aneurysm	1.32 (1.05, 1.65)	.03
18	Dementia	1.48 (1.07, 2.04)	.02	Epilepsy	1.62 (1.48, 1.78)	<.01	Paralysis	1.27 (1.07, 1.50)	.01
19	TIA	1.36 (1.19, 1.55)	<.01	Chronic kidney disease	1.59 (1.39, 1.84)	<.01	TIA	1.26 (1.06, 1.49)	.01
20	Arrythmia	1.36 (1.31, 1.42)	<.01	Thyroid disorder	1.58 (1.53, 1.63)	<.01	Connective tissue disease	1.24 (1.13, 1.35)	<.01
21	Asthma	1.35 (1.32, 1.38)	<.01	Asthma	1.56 (1.52, 1.60)	<.01	Anxiety	1.22 (1.12, 1.33)	<.01
22	Connective tissue disease	1.34 (1.27, 1.41)	<.01	TIA	1.52 (1.24, 1.85)	<.01	COPD	1.16 (1.08, 1.24)	<.01
23	Peptic ulcer disease	1.32 (1.14, 1.52)	<.01	Connective tissue disease	1.39 (1.31, 1.48)	<.01	Asthma	1.14 (1.10, 1.17)	<.01
24	Endometriosis	1.32 (1.24, 1.40)	<.01	Arrythmia	1.39 (1.30, 1.48)	<.01	Chronic respiratory disease	1.06 (1.03, 1.09)	<.01
25	Aneurysm	1.26 (1.04, 1.53)	.02	COPD	1.34 (1.25, 1.43)	<.01	Tuberculosis	0.82 (0.72, 0.94)	.01
26	COPD	1.24 (1.18, 1.30)	<.01	Meniere's disease	1.34 (1.15, 1.56)	<.01	Osteoporosis	0.81 (0.72, 0.92)	<.01
27	Anxiety	1.23 (1.16, 1.29)	<.01	Endometriosis	1.32 (1.24, 1.40)	<.01	Parkinson's disease	0.80 (0.66, 0.97)	.04
28	Hearing impairment	1.23 (1.13, 1.34)	<.01	Congenital diseases	1.23 (1.10, 1.36)	<.01	Substance use disorder	0.76 (0.70, 0.83)	<.01
29	Meniere's disease	1.20 (1.06, 1.35)	<.01	Anxiety	1.23 (1.15, 1.32)	<.01	Dementia	1.51 (0.98, 2.33)	.10
30	Congenital diseases	1.14 (1.06, 1.23)	<.01	Multiple sclerosis	1.18 (1.03, 1.36)	.03	Chromosomal abnormalities	1.31 (0.56, 3.11)	.61
31	Chronic respiratory disease	1.11 (1.08, 1.13)	<.01	Chronic respiratory disease	1.16 (1.13, 1.20)	<.01	Cystic fibrosis	1.25 (0.25, 6.26)	.82
32	Solid organ cancers	1.07 (1.03, 1.10)	<.01	Solid organ cancers	1.09 (1.05, 1.14)	<.01	Chronic urinary tract infection	1.18 (0.84, 1.64)	.40
33	Substance use disorder	0.83 (0.77, 0.89)	<.01	Osteoporosis	0.59 (0.55, 0.63)	<.01	Peptic ulcer disease	1.16 (0.97, 1.40)	.14
34	Osteoporosis	0.63 (0.60, 0.67)	<.01	Eating disorder	0.43 (0.30, 0.61)	<.01	Anemia	1.16 (0.86, 1.57)	.39
35	Eating disorder	0.42 (0.30, 0.59)	<.01	Addison disease	1.55 (0.92, 2.62)	.13	Hematological cancers	1.13 (0.98, 1.30)	.13
36	Autism	2.18 (0.60, 7.96)	.27	Chronic pancreatitis	1.50 (0.97, 2.32)	.09	Heart valve disorder	1.08 (0.99, 1.17)	.13
37	Addison disease	1.25 (0.83, 1.90)	.32	Dementia	1.47 (0.90, 2.40)	.15	Congenital diseases	1.07 (0.96, 1.19)	.27
38	Multiple sclerosis	1.12 (1.01, 1.26)	.06	Aneurysm	1.12 (0.75, 1.66)	.63	Solid organ cancers	1.05 (0.99, 1.10)	.07
39	Hematological cancers	1.08 (0.97, 1.21)	.18	Substance use disorder	1.07 (0.93, 1.24)	.41	Multiple sclerosis	1.01 (0.82, 1.23)	.95
40	Chronic pancreatitis	1.08 (0.83, 1.39)	.61	Hearing impairment	1.07 (0.94, 1.22)	.34	Meniere's disease	1.01 (0.84, 1.22)	.94
41	Chronic urinary tract infection	1.06 (0.87, 1.28)	.61	Irritable bowel disease	1.05 (0.96, 1.15)	.37	Irritable bowel disease	0.95 (0.87, 1.04)	.33
42	Heart valve disorder	1.06 (0.99, 1.13)	.09	Heart valve disorder	1.05 (0.96, 1.15)	.34	Chronic pancreatitis	0.93 (0.68, 1.27)	.70
43	Irritable bowel disease	1.00 (0.93, 1.06)	.90	Hematological cancers	1.02 (0.85, 1.22)	.87	Addison disease	0.89 (0.47, 1.71)	.78
44	HIV/AIDS	0.98 (0.79, 1.20)	.84	Chronic urinary tract infection	1.00 (0.79, 1.28)	.98	HIV/AIDS	0.82 (0.66, 1.03)	.12
45	Tuberculosis	0.91 (0.83, 1.01)	.06	Tuberculosis	1.00 (0.88, 1.14)	.97	Autism	0.66 (0.12, 3.67)	.70
46	Cystic fibrosis	0.86 (0.33, 2.26)	.80	Parkinson's disease	0.96 (0.73, 1.25)	.81	Eating disorder	0.38 (0.12, 1.14)	.12
47	Parkinson's disease	0.85 (0.72, 1.00)	.05	Cystic fibrosis	0.69 (0.19, 2.45)	.63	Endometriosis	NA	NA

The ORs (95% CI) were adjusted for sex, age, education level and Townsend Deprivation Index (in fifth), representing the excessive risk of having a specific long-term condition in people with MASLD, compared with people without MASLD. The conditions were ranked by (1) if the corrected *P* < .05 (indicating statistical difference between MASLD and non-SLD groups), and (2) adjusted OR values (indicating association strength), by descending order.

Abbreviations: COPD, chronic obstructive pulmonary disease; HIV, human immunodeficiency virus; IBD, inflammatory bowel disease; IHD, ischemic heart disease; MASLD, metabolic dysfunction–associated steatotic liver disease; PTSD, post-trauma stress disorder; TIA, transient ischemic attack; VTE, venous thromboembolism;

^*a*^ *P* values were corrected for multiple comparisons using the false discovery rate method.

### MASLD, Multimorbidity, and All-cause Mortality

During a median follow-up of 13 years, 36 664 deaths occurred, with 14 910 among people with MASLD. The major causes of death were cancers, circulatory, respiratory, nervous system, and digestive diseases, in both MASLD and non-MASLD cohorts, with cancers, circulatory, respiratory, and nervous diseases responsible for 80% of deaths.

MASLD was positively, but modestly, associated with increased all-cause mortality after adjusting for the number of LTCs (HR 1.16 [1.13, 1.19]), and had a greater impact on females (HR 1.25 [1.20, 1.29]) than on males (1.10 [1.07, 1.13]). When looking at subgroups stratified by the number of LTCs, having MASLD was associated with increased mortality only when the LTC counts were low (<3 in females and <2 in males) (Table 3).

**Table 3.** Hazard ratios (95% CI) for all-cause mortality comparing MASLD to non-SLD

	Non-SLD	MASLD	MASLD v non-SLD
Number of LTCs at baseline	Events/total	Events/total	HR (95% CI)
**Overall**			
0	8955/178 357	5061/64 823	1.27 (1.23, 1.32)
1	6361/85 262	4298/38 312	1.19 (1.14, 1.24)
2	3488/30 658	2678/17 112	1.09 (1.03, 1.15)
3	1584/9159	1469/6581	1.09 (1.01, 1.17)
4	743/2903	739/2536	0.90 (0.81, 1.01)
≥ 5	623/1481	665/1656	0.79 (0.71, 0.89)
Total	21 754/307 820	14 910/131 020	1.16 (1.13, 1.19)
**Females**			
0	4530/112 190	1620/24 056	1.38 (1.30, 1.46)
1	3386/56 848	1564/16 979	1.29 (1.21, 1.37)
2	1821/20 899	995/8301	1.18 (1.09, 1.28)
3	820/6200	561/3362	1.12 (1.00, 1.26)
4	369/1845	253/1209	0.91 (0.77, 1.08)
≥ 5	283/883	268/838	0.88 (0.74, 1.05)
Total	11 209/198 865	5261/54 745	1.25 (1.20, 1.29)
**Males**			
0	4425/66 167	3441/40 767	1.21 (1.16, 1.27)
1	2975/28 414	2734/21 333	1.12 (1.06, 1.18)
2	1667/9759	1683/8811	1.02 (0.95, 1.09)
3	764/2959	908/3219	1.02 (0.92, 1.13)
4	374/1058	486/1327	0.91 (0.79, 1.05)
≥ 5	340/598	397/818	0.72 (0.61, 0.84)
Total	10 545/108 955	9649/76 275	1.10 (1.07, 1.13)

The Cox model was stratified by region and age group, adjusted for sex, ethnicity, education, Townsend Deprivation Index (in fifths), physical activity, smoking status, weekly alcohol consumption, and long-term condition count. For the sex-mixed model, the model was additionally adjusted for sex.

Abbreviations: LTC, long-term condition; MASLD, metabolic dysfunction–associated steatotic liver disease.

As expected, having more LTCs was associated with increased mortality: compared with people with no LTCs, the HR in those having ≥2 LTCs was 2.18 (2.10, 2.27) in the MASLD cohort and 2.42 (2.34, 2.50) in the non-MASLD cohort. In those having ≥5 LTCs, the HR was 4.26 (3.92, 4.63) and 6.30 (5.80, 6.84), respectively (Fig. S2, Table S3 (31)). The HR per additional LTC was slightly higher in those without MASLD than in those with (1.38 [1.37, 1.40] vs 1.30 [1.29, 1.31]). Sensitivity analyses excluding the first 2 years of follow-up and additionally adjusting for the 5 MASLD-defining cardiometabolic risk factors showed similar results to the primary analysis. When LTC count was modeled as a time-varying variable, we observed qualitatively consistent but slightly stronger associations with mortality than primary analysis. For instance, the adjusted HR estimates per additional LTC were 1.45 (1.44, 1.45) in people without SLD and 1.38 (1.37, 1.38) in people with MASLD (Table S4 (31)).

Sensitivity analysis using HSI to define liver steatosis yielded consistent findings (Table S5 (31)). Among all participants with available data on FLI and HSI, the FLI definition identified 131 020 participants with MASLD, while the HSI definition identified 163 423. Notably, 85% of MASLD cases identified by FLI were also classified as MASLD by HSI. Under the alternative definition, the baseline prevalence of multimorbidity was 13.9% in individuals without MASLD and 20.5% in those with MASLD, closely aligning with estimates from the primary analysis. When examining the association between MASLD and mortality stratified by LTC count, the HR estimates were 1.21 (1.17, 1.25), 1.14 (1.09, 1.18), 1.06 (1.01, 1.12), 1.03 (0.95, 1.11), 0.92 (0.82, 1.03), and 0.90 (0.80, 1.02) for people with 0, 1, 2, 3, 4, and ≥5 LTCs, respectively. The HRs per additional LTC were 1.38 (1.36, 1.39) in people without MASLD and 1.31 (1.30, 1.32) for those with MASLD. These findings closely mirror the primary results using FLI alone to define MASLD, reinforcing the robustness of our conclusion across different case definitions.

Subgroup analyses stratified by age groups, deprivation, education level, smoking status, physical activity, and BMI levels also showed consistently positive association between the number of LTCs and all-cause mortality (Fig. S3 (31)). The HR estimates were similar in people with and without diabetes, at 1.28 (1.26, 1.30) and 1.29 (1.27, 1.32), respectively. The HR estimates were also similar across FIB4 score levels, at 1.28 (1.26, 1.30), 1.32 (1.30, 1.35), and 1.29 (1.23, 1.36) for low, intermediate, and high FIB4 scores, respectively.

### LTCs, Cardiometabolic Risk Factors, and All-Cause Mortality

In people with MASLD, hypertension, diabetes, and low HDL cholesterol among all cardiometabolic risk factors were positively associated with all-cause mortality, with HRs 1.09 (1.04, 1.13), 1.46 (1.41, 1.51), and 1.18 (1.14, 1.22), respectively. Only 16 of the 47 prevalent LTCs were associated with increased mortality. These LTCs included heart failure (2.57 [2.06, 3.22]), stroke (1.61 [1.40, 1.85]), heart valve disorder (1.49 [1.22, 1.82]), IHD (1.33 [1.23, 1.43]), arrythmia (1.21 [1.07, 1.38]), CKD (2.37 [1.91, 2.94]), extrahepatic solid organ cancer (1.70 [1.54, 1.88]), hematological cancers (2.99 [2.29, 3.90]), and COPD (1.41 [1.23, 1.63]), with Parkinson disease showing the highest HR (6.09 [4.47, 8.29]) (Table 4).

**Table 4.** Association between having specific cardiometabolic risk factors and long-term conditions at baseline with all-cause mortality in people with MASLD

	Event/total in those with the condition	HR (95% CI)	*P* value*^a^*
**Cardiometabolic factors**			
Obesity	14 744/129 553	0.96 (0.82, 1.11)	.56
Hypertension	12 346/105 177	1.09 (1.04, 1.13)	<.01
Diabetes	6961/40 909	1.46 (1.41, 1.51)	<.01
High triglyceride	10 833/95 116	0.96 (0.92, 0.99)	.02
Low HDL cholesterol	7680/61 157	1.18 (1.14, 1.22)	<.01
**Conditions for multimorbidity**			
Ischemic heart disease	2870/10 959	1.33 (1.23, 1.43)	<.01
Solid organ cancers	1543/6524	1.70 (1.54, 1.88)	<.01
Arrythmia	1155/4420	1.21 (1.07, 1.38)	.01
COPD	969/3166	1.41 (1.23, 1.63)	<.01
Stroke	797/2911	1.61 (1.40, 1.85)	<.01
Epilepsy	296/1718	1.31 (1.05, 1.64)	.04
Heart valve disorder	423/1546	1.49 (1.22, 1.82)	<.01
Vision impairment	380/1263	1.71 (1.42, 2.06)	<.01
Substance use disorder	321/1091	1.79 (1.42, 2.24)	<.01
Heart failure	514/1067	2.57 (2.06, 3.22)	<.01
Chronic kidney disease	371/971	2.37 (1.91, 2.94)	<.01
Hematological cancers	193/512	2.99 (2.29, 3.90)	<.01
Multiple sclerosis	64/432	1.77 (1.14, 2.74)	.03
Parkinson disease	117/235	6.09 (4.47, 8.29)	<.01
Aneurysm	82/191	3.20 (1.99, 5.12)	<.01
Dementia	23/69	4.42 (2.13, 9.17)	<.01
Asthma	2148/16 770	0.85 (0.78, 0.93)	<.01
Chronic respiratory disease	1678/13 906	0.79 (0.72, 0.87)	<.01
Thyroid disorder	1056/8433	0.82 (0.73, 0.92)	<.01
Endometriosis	96/1598	0.60 (0.40, 0.88)	.03

The table shows the associations between all-cause mortality and cardiometabolic risk factors and long-term conditions that had significant associations in people with or without MASLD, based on false discovery rate–corrected *P* values. For cardiometabolic risk factors, the reference group was people who did not have the risk factor under examination. For long-term conditions, the reference group was people without zero long-term condition. Models are stratified by region and age group (<50, 50-60, ≥ 60 years), adjusted for sex, ethnicity, education, Townsend deprivation index (in fifths), physical activity level, smoking status, alcohol drinking, and number of long-term conditions.

Abbreviations: COPD, chronic obstructive pulmonary disease; HDL, high-density lipoprotein; MASLD, metabolic dysfunction–associated steatotic liver disease.

^*a*^ *P* values were false discovery rate–corrected for multiple comparison.

## Discussion

This study provides insights into the prevalence and impact of extrahepatic LTCs and multimorbidity among individuals with MASLD compared with those without. Our findings revealed a higher burden of multimorbidity in the MASLD population, particularly with higher prevalences of metabolic, cardiovascular, cancer, renal, and mental/behavioral conditions. We also observed positive associations between multimorbidity and all-cause mortality in both cohorts. The association between MASLD and all-cause mortality was dependent on the number of LTCs, with stronger associations in those with lower number of LTCs. LTCs varied in their associations with all-cause mortality. Diabetes, extrahepatic cancers, cardiovascular diseases, and CKD showed strong associations, suggesting they should be the target conditions for primary prevention of multimorbidity and optimization.

Patients with MASLD exhibited a notably higher prevalence of extrahepatic multimorbidity than their non-MASLD counterparts. Specifically, 14.4% of non-MASLD individuals had ≥2 LTCs compared with 21.3% among MASLD individuals, which would further rise to 50.5% if MASLD were counted as an LTC, and to 100.0% if cardiometabolic risk factors were counted. In the non-MASLD cohort, if cardiometabolic risk factors were counted, the prevalence of multimorbidity would rise to 71.6%. We found that MASLD status increased the odds of having a wide spectrum of LTCs, particularly with endocrine/metabolic diseases (including diabetes, gout, thyroid disorder), cardiovascular diseases (hypertension, heart failure, venous thromboembolism, IHD, stroke, etc.), extrahepatic cancers, CKD, mental/behavioral disease (post-traumatic stress disorder, bipolar disorder, depression, anxiety, etc.), and respiratory diseases (asthma, COPD, chronic respiratory disease, etc.), which aligns with previous research (17, 19, 38-41).

The mechanisms for these associations may vary from condition to condition, but obesity and the complex interplay between hepatic lipid accumulation and systemic metabolic disturbances provide a foundation for MASLD's broad associations with multiple LTCs. Insulin resistance, a typical feature of type 2 diabetes, impairs hepatic lipid metabolism, contributing to dysfunction (42). This metabolic disruption promotes excess lipid accumulation in hepatocytes, chronic liver inflammation, and atherogenic dyslipidemia. Altered gut microbiota, or gut dysbiosis, and the evolution of certain pathobionts further exacerbate this inflammatory milieu. Moreover, chronic inflammation within the liver extends its impact by activating pathways like NF-κB, reducing adiponectin while increasing leptin levels, which are implicated in the pathogenesis of cancers (43). Recent genome-wide association analyses have also demonstrated that some underlying genetic variants for MASLD have pleiotropic effects on cardiometabolic and inflammatory traits, including insulin resistance, type 2 diabetes, triglycerides, low-density lipoprotein, and obesity (44-46).

We found that MASLD and multimorbidity were both positively associated with all-cause mortality. On average, each additional LTC was associated with 30% and 38% higher mortality in MASLD and non-MASLD populations, respectively, which was similar in males and females, consistent with previous findings (10, 12). Having MASLD was associated with 16% higher mortality, consistent with previous meta-analysis (47) and recent investigations using the UK Biobank (5, 6). However, we found that the association between MASLD and mortality was dependent on the LTC counts, with a stronger association in people with lower LTC counts. More specifically, having MASLD was associated with 10% to 27% higher mortality for people, combining the sexes, with 0 to 3 LTCs, whereas no additional effect on mortality was found for those with 4 LTCs. This may suggest that people with ≥4 LTCs experience a saturation effect, where baseline mortality risk is already substantially elevated, and the additional contribution of MASLD may be comparatively small, given its slow progression. This was consistent with a previous investigation in the United States (48), which reported the presence of multiple conditions attenuated the association between NAFLD and mortality.

The association between MASLD and mortality was stronger in females than in males (HRs 1.25 vs 1.10), consistently observed within each LTC count. This sex disparity may be attributed to greater differences in prevalences of common and mortality-associated LTCs between MASLD and non-MASLD females than for males. For instance, the ORs were higher in females than in males for IHD, stroke, diabetes, COPD, and heart failure, while significantly higher ORs were present with some LTCs in females, but not in males, such as extrahepatic cancers and multiple sclerosis (Table 2).

Our analysis highlights that extrahepatic cancers, diabetes, CKD, COPD, and cardiovascular diseases (heart failure, IHD, stroke, etc.) significantly contribute to increased mortality risk. This emphasizes the substantial potential for reducing mortality via primary prevention and/or aggressive management of these key coexisting conditions, with large benefit of mortality reduction. The high burden of multimorbidity in people with and without MASLD calls for integrated care approaches that address multiple comorbid conditions simultaneously (49). Given the progressive nature of MASLD and its close association with metabolic and cardiovascular conditions, routine screening for these comorbidities should be an integral part of MASLD management (50, 51). Lifestyle modifications and risk factor management are recommended public health interventions to reduce the incidence and progression of MASLD, thereby potentially mitigating the broader burden of multimorbidity and associated mortality (51). The recent introduction of GLP1RAs (52) will likely revolutionize the treatment of MASLD and many of its comorbid conditions. It will be important to ensure equal access to these powerful medications.

Although menopause status is an established risk factor for various health outcomes in females, we did not include it in our analysis for several reasons. First, at baseline, a high proportion of women had already reported being postmenopausal (71.8% in non-SLD and 63.5% in MASLD). Second, the UK Biobank is a middle-aged cohort with a mean baseline age of 56.4 years old for females, and by the end of follow-up 97.3% females were aged ≥55 years and 100% ≥45 years—ages by which most women have undergone menopause. Third, the UK Biobank does not have data for the age of experiencing menopause, and the timing of menopause during follow-up was unknown for women who were premenopausal at baseline. Additionally, age also acts as a reasonable proxy for menopausal status in females.

We performed analyses on the associations between cardiometabolic risk factors, LTCs, and mortality, as shown in Table 4. However, it is important to emphasize that in individuals with multimorbidity, overlapping and interacting LTCs may jointly contribute to mortality, making it difficult to isolate the effect of individual conditions. Therefore, the associations observed should be interpreted as estimates of risk rather than causal attribution.

This study provides a more detailed exploration of how multimorbidity differed in people with and without MASLD, and the interactions between MASLD and multimorbidity by considering a wide array of LTCs and their specific contributions to mortality. Nevertheless, several limitations should be acknowledged. First, we used FLI to evaluate steatosis, instead of more accurate imaging techniques, which are the current gold standard. However, previous evidence has shown that FLI yielded high accuracy (6) and was more accurate than other steatosis indices (53) when compared with imaging-based assessment. In the sensitivity analysis using the HSI to identify liver steatosis, we observed similar results to the primary results using the FLI, reinforcing the robustness of our conclusion. Having said that, future research using biopsy or imaging approaches for MASLD case identification is warranted. Second, the UK Biobank population is a predominantly White cohort with higher socioeconomic status and health status than the general UK population; therefore, the prevalence estimates may not be representative of the whole UK population (54). This may limit the generalizability of our findings to populations of different ethnic or socioeconomic backgrounds. Third, we replied on self-reported data for lifestyle factors, including physical activity, smoking, and alcohol consumption, which may be susceptible to information biases. Particularly, inaccuracies in self-reported alcohol consumption may result in misclassification of excessive alcohol drinking, potentially obscuring overlap between MASLD and alcohol-related liver disease, and, consequently, affecting the accurate identification of MASLD cases. Fourth, the primary exposure was the number of LTCs at baseline, and future studies are warranted to investigate specific LTC clusters. Fifth, we did not consider detailed follow-up on medical treatment and intervention in the analysis, which was not available in the UK Biobank dataset.

In conclusion, this study revealed that individuals with MASLD had a substantially higher burden of multimorbidity than those without MASLD, particularly in relation to diabetes, extrahepatic cancer, COPD, CKD, and cardiovascular diseases. It also elucidated the interplay between MASLD and multimorbidity on all-cause mortality. Having 1 additional LTC was associated with 30% higher mortality risk, whereas for the same LTC counts, the association between additionally having MASLD and all-cause mortality was more pronounced in those with low number of LTCs. Therefore, addressing multimorbidity in patients with MASLD through multidisciplinary and proactive management of multimorbidity is crucial to improving patient outcomes and reducing the overall public health impact of MASLD.

## Data Availability

UK Biobank data are available to registered researchers at https://www.ukbiobank.ac.uk/. Analytic codes are available upon request.

## Abbreviations

BMI: body mass index
BP: blood pressure
CKD: chronic kidney disease
COPD: chronic obstructive pulmonary disease
FLI: fatty liver index
HbA1c: glycated hemoglobin
HDL: high-density lipoprotein
HSI: hepatic steatotic index
IHD: ischemic heart disease
LTC: long-term condition
MASLD: metabolic dysfunction–associated steatotic liver disease
NAFLD: nonalcoholic fatty liver disease
TG: triglyceride
